# Screening for known mutations in *EIF2B *genes in a large panel of patients with premature ovarian failure

**DOI:** 10.1186/1472-6874-4-8

**Published:** 2004-10-26

**Authors:** Anne Fogli, Fernande Gauthier-Barichard, Raphael Schiffmann, Vien H Vanderhoof, Vladimir K Bakalov, Lawrence M Nelson, Odile Boespflug-Tanguy

**Affiliations:** 1 INSERM UMR 384, Faculté de Médecine, 28 place Henri Dunant BP 38, 63001 Clermont-Ferrand cedex, France; 2 Developmental and Metabolic Neurology Branch, National Institute of Neurological Disorders and Stroke, National Institutes of Health, Bethesda, Maryland, USA; 3 National Institute of Child Health and Human Development, National Institutes of Health, Bethesda, Maryland, USA

## Abstract

**Background:**

Premature Ovarian Failure (POF), defined as the development of hypergonadotropic amenorrhea before the age of 40 years, occurs in about 1% of all women. Other than karyotype abnormalities, very few genes are known to be associated with this ovarian dysfunction. Recently, in seven patients who presented with POF and white matter abnormalities on MRI (ovarioleukodystrophy) eight mutationswere found in *EIF2B2*, *4 *and *5*.

**Methods:**

To further test the involvement of known mutations of *EIF2B *genes in POF, we screened 93 patients with POF who did not have identified leukodystrophy or neurological symptoms. We evaluated these eight mutations and two additional mutations that had been found in patients with milder forms of eIF2B-related disorders. We used restriction enzymes and direct sequencing.

**Results:**

None of the known mutations in *EIF2B *genes, either homozygous or heterozygous, were identified in our 93 patients with pure 46,XX POF. The upper 95 % confidence limit of the proportion 0/93 is 3.2%.

**Conclusions:**

We conclude that eIF2B mutations, already described in cases of POF associated with white matter abnormalities, are an uncommon cause of pure spontaneous premature ovarian failure.

## Background

Premature Ovarian Failure (POF) can present as a primary or secondary amenorrhea and is associated with elevated gonadotropins before 40 years of age. POF affects 1% of all women and occurs in 0.1% before the age of 30 years [1]. POF has been associated with karyotype abnormalities, including various X chromosome aberrations such as Turner syndrome, which causes depletion of ovarian follicles during development [2]. While conditions such as autoimmune diseases are also associated with POF, the cause is unknown in about 90 % of cases. However, since many affected women have a family history of the condition, predisposition to POF may be inherited [3]. To date, mutations associated with POF have been identified in a small number of genes [4], including those encoding the inhibin alpha [5], the FSH receptor [6], the LH/choriogonadotrophin receptor [7], and the forkhead transcription factor 2 [8]. No more than 10% of women with ovarian failure have mutations in these different genes [8].

Recently three of the five *EIF2B *genes (*EIF2B2*, *4 *and *5*) were reportedly involved in seven patients who presented with POF and white matter abnormalities on MRI (ovarioleukodystrophy) [9]. These genes encode the five subunits of the eucaryotic initiation factor 2B (eIF2B alpha to epsilon), which is involved in the first step of protein synthesis. eIF2B-related disorders include a large group of phenotypes with a recognizable MRI pattern but different clinical severities. The clinical *spectrum *can range from a rapid course leading to death in severe congenital forms to asymptomatic MRI findings in adult patients [10,11]. Ovarioleukodystrophy might present in a phase without neurological symptoms and an apparently isolated form of POF [9]. Therefore, we screened a series of 93 patients with apparently pure, karyotypically normal POF for mutations in *EIF2B *genes.

## Methods

### Selection of patients with premature ovarian failure

In the current study, we evaluated the presence of *EIF2B *mutations in 93 unrelated and well-characterized women with POF. An institutional review board approved the study and all participants gave a written informed consent. Referring physicians made the diagnosis of premature ovarian failure based on the following criteria: development of at least 4 months of amenorrhea before age 40 associated with two serum FSH levels in the menopausal range. Women with premature ovarian failure as a result of surgery, radiation, chemotherapy, or known karyotype abnormalities were not included in the study. There were 6 Asians, 12 Blacks, 4 Hispanics and 71 Caucasians. The median age at the onset of menstrual irregularity was 24.5 years (range 13 to 39). Eighteen women had a family history of POF. All women underwent a history and physical examination and laboratory screening to confirm the diagnosis of POF and all had a normal karyotype. None of the women had evidence of a neurological disorder.

### EIF2B mutations screening

Genomic DNA was extracted from peripheral blood using standard procedures.

The exons of the genes *EIF2B2*, *4 *and *5 *which contain mutations found in POF patients or in milder forms of eIF2B-related disorders were amplified by the polymerase chain reaction (PCR) as previously described (Table 1)[11].

**Table 1:** Sequences of PCR primers used and their PCR conditions.

Nucleotide change tested (gene)	Primers sequences (5'-3')	PCR conditions
C512T (*EIF2B2*), C547T (*EIF2B2*)	F: GCAAAACCGTTCTTAC R: CCTACCCATCTCTCGTTTAT	PCR preparation: 1.5 mM MgCl2, 0.225 mM dNTP, 0.8 μM primers, 100 ng primers, 1 unit AmpliTaq Gold™(Applied Biosystems), 1X Taq buffer.
607–612del/insTG (*EIF2B2*), A638G (*EIF2B2*)	F: GGAAATTATGTGCTGGATATG R: ACTTTATTCTCTCACCGTGGAT	
P243L (*EIF2B4*)	F: ATGCTCAAGCTCCCTTTCAA R: CTTCACAACTTACAAAGCCT	
R374C (*EIF2B4*)	F: ATTCAAGCACCTGGCATGAT R: CGCTGCACTCCATCCTTATC	PCR reaction: 95°C 12 min, 35 cycles (94°C 30 s, 55°C 30 s, 72°C 45 s), 72°C 10 min, 4°C.
T1393C (*EIF2B4*), T1465C (*EIF2B4*)	F: TGTCCTGTAAGTAGGGGACCTT R: AAGGGGTTGTGAAGTCTGGA	
G338A (*EIF2B5*), C583T (*EIF2B5*)	F: GAGAAGGACTGTGAGTGCTGA R: GCCTTCTAAGGGGACAATAAC	

F: forward primer, R: reverse primer

Nine mutations were tested by restriction enzymes directly on PCR products (Table 2): 500 ng of PCR products were incubated with 1 unit of specific restriction enzyme from Biolabs^® ^Inc. for 90 minutes, according to the supplier's instructions. Restriction fragments were analyzed by standard acrylamide gel electrophoresis.

**Table 2:** *EIF2B2*, *4 *and *5 *mutations tested with restriction enzymes.

Mutation tested: nucleotides changes (amino acid changes)	Mutated gene	Restriction enzyme used	PCR product size (base pairs)	Restriction profile (number of restriction fragments: their size in base pairs)		
				
				No mut*	Het mut*	Hom mut*
C512T (S171F)	*EIF2B2*	Hpy188III	251	4 fragments: 51, 29, 16 and 155 bp.	5 fragments: 51, 29, 16, 155 and 171 bp.	3 fragments: 51, 29 and 171 bp.
607–612del/insTG (M203fs)	*EIF2B2*	HphI	313	2 fragments: 310 and 3 bp.	4 fragments: 310, 142, 164 and 3 bp.	3 fragments: 142, 164 and 3 bp.
C547T (R183stop)	*EIF2B2*	EcoNI	253	2 fragments: 120 and 133 bp.	4 fragments: 120, 133, 14 and 119 bp.	3 fragments: 120, 14 and 119 bp.
A638G (E213G)	*EIF2B2*	BsmAI	313	2 fragments: 153 and 160 bp.	3 fragments: 313, 160 and 153 bp.	1 fragment: 313 bp
P243L (C728T)	*EIF2B4*	AciI	612	3 fragments: 353, 223 and 36 bp.	4 fragments: 576, 353, 223 and 36 bp.	2 fragments: 576 and 36 bp.
R374C (C1120T)	*EIF2B4*	HpyCH4IV	640	2 fragments: 447 and 193 bp.	3 fragments: 640, 447 and 193 bp.	1 fragment: 640 bp.
T1393C (C465R)	*EIF2B4*	BsrDI	694	3 fragments: 368, 32 and 294 bp.	4 fragments: 368, 32, 294 and 326 bp.	2 fragments: 368 and 326 bp.
T1465C (Y489H)	*EIF2B4*	NlaIII	707	3 fragments: 109, 310 and 288 bp.	5 fragments: 109, 310, 288, 57 and 231 bp.	4 fragments: 109, 310, 57 and 231 bp.
G338A (R113H)	*EIF2B5*	Fnu4HI	800	3 fragments: 115, 633 and 52 bp.	4 fragments: 115, 633, 52 and 748 bp.	2 fragments: 748 and 52 bp.

* No mut: no mutation; Het mut: heterozygous mutation; Hom mut: homozygous mutation.

The C583T (R195C) mutation in the *EIF2B5 *gene was tested by direct sequencing of exon 4 as previously described [11].

## Results

None of the eight mutations already described in ovarioleukodystrophy were detected in our 93 patients with pure 46,XX POF, neither in a homozygous nor in a heterozygous state. In addition, the mutations C728T and C1120T (*EIF2B4*) described in milder forms of eIF2B-related disorders were not found in this series of 93 patients with POF. The upper 95 % confidence limit of the proportion 0/93 is 3.2%.

## Discussion

eIF2B-related disorders include a large group of phenotypes with different clinical severities. Individuals can be classified into three clinical groups according to their age at disease onset: <2 years (group 1), 2 to 5 years (group 2) and > 5 years (group 3) [11]. Group 3 corresponds to individuals with the milder form of the disease, including the six families (seven patients) already described presenting with ovarioleukodystrophy [9].

In these six eIF2B-mutated families, neurological symptoms with abnormalities of the cerebral white matter on MRI were associated with primary or secondary amenorrhea due to POF [9]. A correlation was observed between the age of onset of the neurological deterioration and the severity of the ovarian failure, suggesting a common pathophysiological pathway [9].

The mutated eIF2B may be responsible for both increased apoptosis of ovarian follicles leading to POF, and a defect in glial cell development causing abnormal formation of white matter structures. In ovarioleukodystrophy, a phase of amenorrhea without neurological symptoms can be observed, suggesting that an apparently isolated case of POF might be due to *EIF2B *mutations. In the present study, we tested for *EIF2B *mutations a series of 93 patients with pure, karyotypically normal POF without identified signs of cerebral dysfunction.

In eIF2B-related disorders, a correlation exists between genotype and disease onset [11]. The mutations G338A (*EIF2B5 *gene) and A638G (*EIF2B2 *gene) are found in 71% of families with late onset forms of eIF2B-related disorders (group 3) [11]. In ovarioleukodystrophy, 4/6 families have a G338A or A638G mutation in a heterozygote or a homozygote state. Thus, to further evaluate involvement of eIF2B mutations in apparently isolated cases of POF, we restricted our screening to the 10 mutations associated with the late onset form (group 3) of eIF2B-related disorders. In the present series of 93 patients with pure, karyotypically normal POF, no mutations were detected, suggesting a low frequency of *EIF2B *mutations in women with POF who have no apparent neurological signs.

## Conclusions

For patients presenting with POF without neurological signs or MRI abnormalities, the routine screening of the *EIF2B *mutations is not clinically indicated.

## Competing interests

The author(s) declare that they have no competing interests.

## Authors'contributions

AF and FGB carried out the molecular genetics studies, including enzyme restrictions (AF) and sequencing (AF and FGB). AF drafted and conceived of the study. RS participated in the coordination of the study. VHV, VKB, LMN recruited and evaluated the patients, collected DNA samples, participated in the design and coordination of the study, and helped in drafting the manuscript. OBT conceived of the study, and participated in its design and coordination. All authors participated in the writing of the manuscript and have read and approved the final manuscript.

## Pre-publication history

The pre-publication history for this paper can be accessed here:


